# The role of surgeon volume on patient outcome in total knee arthroplasty: a systematic review of the literature

**DOI:** 10.1186/1471-2474-13-250

**Published:** 2012-12-14

**Authors:** Rick L Lau, Anthony V Perruccio, Rajiv Gandhi, Nizar N Mahomed

**Affiliations:** 1 Division of Orthopaedics, Department of Surgery Kingston General Hospital, Queen’s University, 76 Stuart St., Nickle 3, Rm 9-309, Kingston, ON, K7L 2V7, Canada; 2 Division of Orthopaedic Surgery and The Arthritis Program Toronto Western Hospital, University Health Network Institute of Health Policy, Management and Evaluation, University of Toronto, 399 Bathurst St., EW 1-427, Toronto, ON, M5T 2S8, Canada; 3 Division of Orthopaedics, Department of Surgery Toronto Western Hospital, University Health Network, University of Toronto, 399 Bathurst St., EW 1-439, Toronto, ON, M5T 2S8, Canada

**Keywords:** Surgeon volume, Knee arthroplasty

## Abstract

**Background:**

A number of factors have been identified as influencing total knee arthroplasty outcomes, including patient factors such as gender and medical comorbidity, technical factors such as alignment of the prosthesis, and provider factors such as hospital and surgeon procedure volumes. Recently, strategies aimed at optimizing provider factors have been proposed, including regionalization of total joint arthroplasty to higher volume centers, and adoption of volume standards. To contribute to the discussions concerning the optimization of provider factors and proposals to regionalize total knee arthroplasty practices, we undertook a systematic review to investigate the association between surgeon volume and primary total knee arthroplasty outcomes.

**Methods:**

We performed a systematic review examining the association between surgeon volume and primary knee arthroplasty outcomes. To be included in the review, the study population had to include patients undergoing primary total knee arthroplasty. Studies had to report on the association between surgeon volume and primary total knee arthroplasty outcomes, including perioperative mortality and morbidity, patient-reported outcomes, or total knee arthroplasty implant survivorship. There were no restrictions placed on study design or language.

**Results:**

Studies were variable in defining surgeon volume (‘low’: <3 to <52 total knee arthroplasty per year; ‘high’: >5 to >70 total knee arthroplasty per year). Mortality rate, survivorship and thromboembolic events were not found to be associated with surgeon volume. We found a significant association between low surgeon volume and higher rate of infection (0.26% - 2.8% higher), procedure time (165 min versus 135 min), longer length of stay (0.4 - 2.13 days longer), transfusion rate (13% versus 4%), and worse patient reported outcomes.

**Conclusions:**

Findings suggest a trend towards better outcomes for higher volume surgeons, but results must be interpreted with caution.

## Background

Primary total knee arthroplasty (TKA) is recognized as an effective treatment for alleviating the pain and disability associated with end stage knee osteoarthritis [1,2]. Despite the widespread success of primary TKA, a significant minority of patients (ranging from 10-20%) continue to experience complications and report poor outcomes following this procedure [2,3]. The implications are significant considering that the total number of primary TKA procedures is expected to increase by over 600% in the United States alone, to nearly 3.5 million procedures over the next 20 years [4].

A number of factors have been identified as influencing TKA outcomes, including patient factors such as gender and medical comorbidity [5,6], technical factors such as alignment of the prosthesis [7], and provider factors such as hospital and surgeon procedure volumes [8-10]. Recently, strategies aimed at optimizing provider factors have been proposed, including regionalization of total joint arthroplasty to higher volume centers, and adoption of volume standards [8,10,11].

Higher hospital procedure volumes have been associated with improved outcomes across a variety of cardiac, vascular and oncologic surgeries [12-14]. Several studies have examined the influence of hospital volume on TKA outcomes as well [10,15-17]. Higher hospital volumes have been shown to be negatively associated with mortality rates and positively associated with implant survivorship following TKA [18]. However, the association between surgeon volume and TKA outcomes is unclear. Over the past two decades a number of studies have examined the influence of surgeon volume on various TKA outcomes. However, there has not been a systematic review which has focused exclusively on surgeon volume and primary TKA outcomes. To contribute to the discussions concerning the optimization of provider factors and proposals to regionalize TKA practices, we undertook a systematic review to investigate the association between surgeon volume and primary TKA outcomes.

## Methods

### Eligibility criteria

To be included in the review, the study population had to include patients undergoing primary TKA. Eligible studies had to compare primary TKA outcomes between low volume surgeons and high volume surgeons. Primary TKA outcomes including perioperative mortality and morbidity, patient-reported outcomes, or TKA implant survivorship were examined. Relevant papers were reviewed using the Preferred Reporting Items for Systematic reviews and Meta Analyses (PRISMA) statement as a guideline [19].

### Information sources and study selection

We searched the Pubmed, OVID MEDLINE (1966 to 2011), and EMBASE (1974-2011) databases in October 2012 using the following search criteria: (knee arthroplasty OR knee replacement) AND (surgeon volume OR surgeon experience). No restrictions were placed on publication date, study design or language. Following these initial searches, we performed a second electronic search using the following terms: arthroplasty and volume, joint replacement and volume to further the breadth of our search. Two authors screened all titles and abstracts retrieved from this search (RLL, RG). Full-length articles were retrieved for all articles that were considered either potentially relevant or where there was uncertainty as to the relevance. Additionally, the bibliographies of these full-length articles were reviewed and full length articles retrieved for those references deemed potentially relevant or of uncertain relevance. Full-length articles were reviewed by two reviewers (RLL,RG) and data was independently extracted. When manuscripts included data on TKA and total hip arthroplasty (THA), or primary TKA and revision TKA, they were excluded unless the primary TKA data were reported separately. Similarly, when manuscripts included data on hospital and surgeon volume they were excluded unless the surgeon volume data was reported separate from hospital volume data.

### Data extraction

The following study characteristics were documented: study design, date, data source, sample size, characterization of surgeon volume, and examined primary TKA outcome(s).

### Quality appraisal of studies

The studies that were included in our review underwent an appraisal of the quality of methodology as outlined by the Grading of Recommendations Assessment, Development and Evaluation (GRADE) system [20].

## Results

### Study selection

**Figure 1:**
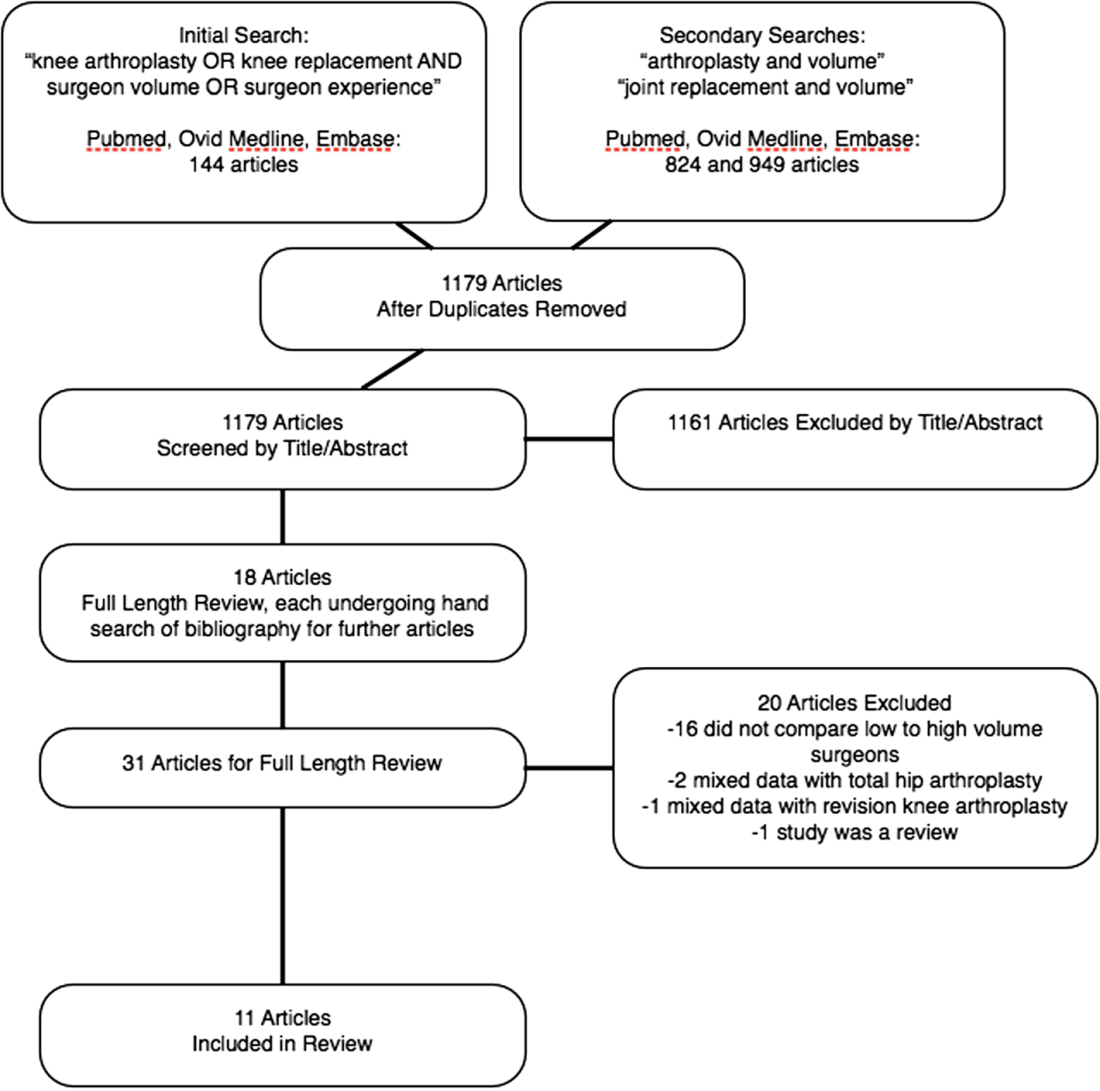
Selection of articles for review.

We identified 144 abstracts using our initial electronic search for screening. Secondary electronic searches identified 824 and 949 abstracts for screening. After title and abstracts were screened, 18 met inclusion criteria and the full-length articles were retrieved and reviewed [8-10,18,21-34]. Hand searches of the bibliographies of these 18 articles resulted in the inclusion of 13 more articles [11,15-17,35-43], for a total of 31. Following the full-length reviews, 20 articles were excluded. Sixteen were excluded because they did not compare low volume surgeons to high volume surgeons [11,15-17,29,33,35-44], two studies were excluded because TKA data was mixed with THA data [23,34], one study was excluded because primary TKA data was reported with revision TKA data [10], and one study was found to be a review article and thus, not eligible for inclusion [24]. Eleven studies met our inclusion/exclusion criteria and were retained for this review [8,9,18,21,22,25-28,30,32] (Figure 1).

### Study characteristics

#### Study design

All included studies were non-randomized, comparative (low vs. high surgeon volume) observational trials. Nine of the 11 studies were retrospective, and 2 were prospective (Table 1).

**Table 1:** Studies on surgeon volume and TKA outcome

**Study**	**Patients (n)**	**Study design**	**Country**	**Year data collected**	**Data source**	**Outcomes**
Kreder et al (2003)	14352	Retrospective	Canada	1992-1996	OHIP and CIHI	MR (in hospital), MR (90d), infection, revision, complication, LOS
Katz et al (2004)	80904	Retrospective	US	2000	Medicare	MR (90d), MI, infection, pneumonia, PE
Katz et al (2007)	906	Prospective	US	2000	Medicare, patient survey	WOMAC, patient satisfaction, 90 degree flexion, full extension
Muilwijk et al (2007)	6357	Retrospective	Netherlands	1996-2003	Dutch Nosocomial Infection Surveillance Network	Infection
Manley et al (2009)	53971	Retrospective	US	1997-2004	Medicare	2, 5, 8 year implant survivorship
Ong et al (2009)	NR	Retrospective	US	1997-2004	Medicare	Procedure duration
Yasunaga et al (2009)	3577	Prospective	Japan	2006-2007	Web based surgeon survey	MR (in-hospital), infection, DVT, PE, pneumonia, LOS
Paterson et al (2010)	27217	Retrospective	Canada	2000-2004	OHIP and CIHI	MR (90d), revision, readmission for surgery, LOS, complications
Wei et al (2010)	31618	Retrospective	Taiwan	2000-2003	NHI	LOS, hospital charges, infection, complications
Styron et al (2011)	67713	Retrospective	US	2002	HCUP database	LOS
Baker et al (2011)	260	Retrospective	UK	2006-2007	Local Database and National Joint Registry	Transfusion rate

(*MR* Mortality rate, *LOS* Length of stay, *HCUP* Health Care Utilization Project, *PE* Pulmonary embolus, *DVT* Deep venous thrombosis, *MI* Myocardial infarction, *WOMAC* Western Ontario and McMaster Universities Osteoarthritis Index, *OHIP* Ontario Health Insurance Plan, *CIHI* Canadian Institute for Health Information, *NHI* National Health Insurance, *NR* Not reported).

#### Sample size

Sample size (n) was recorded as number of patients. One study did not report the actual number of TKA procedures in their manuscript [28]. The remaining studies comprised 286 875 patients. Across studies, sample size varied from a minimum of 260 to a maximum of 80 904 (Table 1).

#### Patient data source

The majority of studies were North American based. Five studies were from the United States (US), 2 from Canada, 1 from United Kingdom, 1 from Japan, 1 from Taiwan, and 1 from the Netherlands.

The US studies made use of two databases to identify the patient cohorts. Four of the 5 studies utilized the US Medicare database [8,9,18,28], and one used the Health Care Cost and Utilization Project Nationwide Inpatient Sample (HCUP-NIS) database [21]. The Medicare database contains claims data for services provided to beneficiaries (ages ≥65 years and those with certain disabilities), including demographic, diagnostic and procedural data. The HCUP-NIS database was developed to help track and analyze trends in health care utilization, cost, quality and utilization and is collected on an annual basis from a nationwide sample of community hospitals in the US [10]. It contains information on all patient admissions at the participating hospitals with patient specific demographic and hospital data, as well as discharge information.

The Canadian studies utilized the Ontario Health Insurance Plan (OHIP) and Canadian Institute for Health Information (CIHI) databases [25,32]. OHIP is a government-run health insurance plan. Its database contains claims data for all physician billing (including patient, physician and hospital specific data) in the province of Ontario, Canada’s most populous province. The CIHI maintains a national database of hospital admissions, and was used to supplement the OHIP data in the Kreder et al. and Paterson et al. studies [25,32].

The United Kingdom study utilized a local joint replacement database to identify patients, and used hospital records to collect data [22]. The national joint registry in United Kingdom was also used to collect demographic and procedure related data.

The Japanese study was derived from a nation-wide internet survey of orthopedic surgeons [27]. Surgeons provided patient information to survey questions from patient’s medical records. The data was then compiled into a database and results analyzed.

The Taiwanese study used the National Health Insurance (NHI) database [26]. The NHI stores data on 99% of the population in Taiwan, with patient specific clinical and demographic data collected since 1995.

The Dutch study used the surveillance network for nosocomial infections [Preventie Ziekenhuisinfecties door Surveillance Network database (PREZIES)] [30]. This database was designed in 1996, and collects data from participating hospitals on surgical site infections.

### Defining surgeon volume

In classifying surgeon volume, 10 of the 11 studies used the annual number of procedures performed by the surgeon [8,9,18,21,22,25,26,28,30,32], whereas one study utilized the career life-time number of procedures [27]. Significant variability was noted across studies in the manner in which surgeons were classified as high versus low volume. Thresholds were primarily based on tertiles or quartiles of the study-specific surgeon volume distributions. Five studies chose to use quartiles, and for these we labeled the surgeon volume categories as low, medium, high and very high [8,18,21,25,28]. Four studies chose to define surgeon volume based on tertiles; for these, we used the low, medium and high volume categories [26,27,30,32]. One study defined low volume surgeons as <52 procedures per year [22]. One study defined low volume surgeons as <6 procedures per year [9]. Due to the variability in categorization of surgeon volumes across studies, ‘low’ surgeon volumes ranged from < 3 to < 52 procedures per year across studies, and ‘high’ surgeon volume ranged from > 5 to > 70 procedures per year (Table 2).

**Table 2:** Surgeon volume thresholds and outcomes of the studies

**Study**	**Surgeon volume thresholds**				**Outcomes**	**Result**	**LV vs. HV surgeons**	Adjusted odds ratios* (95% CI)
	**Low**	**Medium**	**High**	**V. High**				
Kreder et al (2003)	<14	14-42	>42	--	LOS	**p < 0.05**	**11.5 vs 10.0 days**	**NR**
					MR (in-hospital)	NS	0.5% vs 0.3%	NR
					MR (90 day)	NS	0.8% vs 0.4%	1.76 (0.8-3.8) (LV:HV)
					Infection (3 yr)	NS	2.1% vs 2.3%	0.88 (0.5-1.3)(LV:HV)
					Revision (3 yr)	NS	2.2% vs 1.9%	1.00 (0.6-1.7)(LV:HV)
					Medical complication	NS	9% vs 11%	0.98 (0.7-1.3)(LV:HV)
Katz et al (2004)	1-12	13-25	26-50	>50	Pneumonia	**p < 0.01#**	**1.68% vs 1.02%**	**0.72 (0.54-0.95) (HV:LV)#**
					Infection	**p < 0.01#**	**0.55% vs 0.29%**	**0.62 (0.37-1.06) (HV:LV)#**
					MI	NS	0.8% vs 0.69%	0.90 (0.64-1.28)(HV:LV)
					PE	NS	0.76% vs 0.74%	1.06 (0.73-1.54)(HV:LV)
					MR (90d)	NS	0.67% vs 0.58%	0.97 (0.66-1.43)(HV:LV)
Katz et al (2007)	1-6		> 6		poor WOMAC score (score < 60)	**p < 0.05**	**22.6% vs 8.4%**	**2.1 (1.1-4.2) (LV:HV)**
					flex to 90 degrees	**p < 0.05**	**NR**	**2.9 (1.6-5.5) (LV:HV)**
					full extension	**p < 0.05**	**NR**	**2.2 (1.1-4.4) (LV:HV)**
					Dissatisfied with TKA	NS	NR	1.4 (0.6-3.3)(LV:HV)
Muilwijk et al (2007)	5	--	12	--	Infection	**p < 0.05**	**4.9% vs 2.1%**	**0.43 (0.23-0.80) (HV:LV)**
Manley et al (2009)	1-12	13-25	26-50	>50	Early - mid term survivoship (8 years)	NS	NR	0.94 (0.78-1.15)(LV:HV)
Ong et al (2009)	1-12	13-25	26-50	>50	Procedure duration	**p < 0.05**	**165 mins vs 135 mins**	**NR**
Yasunaga et al (2009)	Used surgeon career volume of TKR as surgeon volume variable: <100, 100-499, >499				Medical complication	NS	7.7% vs 13.5%	1.17 (0.66-2.07) (HV:LV)
		LOS	NS	38.9 vs 35 days	NR			
Paterson et al (2010)	2-35	36-50	51-70	>70	LOS	**p < 0.05**	**NR**	**NR**
					MR	NS	0.624% vs 0.547%	1.02 (0.63-1.67)(HV:LV)
					Readmission for surgery (1 yr)	NS	0.594% vs 0.403%	0.81 (0.41-1.62)(HV:LV)
					Revision (1 yr)	NS	1.279% vs 0.922%	0.75 (0.51-1.09)(HV:LV)
					Medical Complication	NS	4.217% vs 4.553%	0.90 (0.67-1.19)(HV:LV)
Wei et al (2010)	1-3	4-9	10-463	--	LOS	**p < 0.05**	**10.79 vs 8.66 days**	**NR**
					Infection	**p < 0.05**	**0.99% vs 0.54%**	**2.31 (1.379-3.876) (LV:HV)**
Styron et al (2011)	1-17	18-35	36-66	>67	LOS	**p < 0.05**	**4.14 vs 3.74 days**	**NR**
Baker et al (2011)	1-52		>52		Transfusion rate	**p < 0.05**	**13% vs 4%**	**NR**

(*LV* Low volume, *HV* High or v. high volume, *MR* Mortality rate, *LOS* Length of stay, *NS* Not significant, *NR* Raw proportions not reported, *PE* Pulmonary embolus, *DVT* Deep venous thrombosis, *MI* Myocardial infarction, *mins* Time in minutes, *WOMAC* Western Ontario and McMaster Universities Osteoarthritis Index, * - see Table 3 for covariates adjusted for in adjusted odds ratios, # - significant for trend, 95% CI = 95% confidence intervals, HV:LV = odds ratio expressed as high volume to low volume, *LV*:*HV* Odds ratio expressed as low volume to high volume, *TKA* Total knee arthroplasty).

### Examined outcomes

Across studies, a variety of perioperative outcomes were reported (Table 1), including in-hospital mortality rate (MR) (2 of 11 studies) [27,32], 90 day MR (3 of 11 studies) [8,25,32], pulmonary embolism (PE) (2 of 11 studies) [8,27], and deep venous thrombosis (DVT) (1 of 11 studies) [27]. Surgical site infections were reported in 5 of 11 studies [8,26,27,30,32]. Variable time frames were considered: in-hospital infections [26], 90-day post-operative period [8], 12-month post-operative period [30], and 3-year post-operative period [32]. One study did not specify time point [27]. Two studies measured deep infections [8,27], and 3 studies did not specify [26,30,32].

Postoperative medical complications were reported in 6 of 11 studies [8,22,25-27,32]. Medical complications were defined differently in each study, and included myocardial infarction [8,25,26,32], pneumonia [8,25-27], cerebrovascular accident [25,32], anaesthetic complications [25], urinary tract infection [26], upper gastrointestinal bleeding [26], and anemia [22].

Five studies examined length of stay [21,25-27,32] and one study examined procedure length [28] (Table 1). Three studies examined 1-year, 3-year- and 8-year implant survivorship, respectively [18,25,32], and one study examined patient-reported outcomes, including the Western Ontario and McMaster Universities Osteoarthritis Index (WOMAC), patient satisfaction, patient-reported ability to flex 90 degrees, and patient-reported ability to fully extend [9] (Table 1).

Nine of 11 studies reported data on the measured outcomes using raw comparative proportions as well as adjusted odds ratios (OR) from multivariate logistic regression analyses adjusting for a variety of covariates. A summary of the study-specific covariates are presented in Table 3.

**Table 3:** Confounding variables controlled for in multivariate analysis in each study

**Study**	**Covariates controlled**
Kreder et al (2003)	age, comorbidity, gender, diagnosis, hospital procedure volume
Katz et al (2004)	age, gender, comorbidity, Medicaid eligibility, diagnosis, hospital procedure volume
Katz et al (2007)	age, gender, race, education, diagnosis, income, comorbidity, preoperative patient reported outcome (WOMAC)
Muilwijk et al (2007)	ASA class
Manley et al (2009)	age, gender, race, diagnosis, hospital procedure volume, hospital teaching status, hospital ownership, hospital region, income
Manley et al (2009)	age, gender, race, diagnosis, hospital procedure volume, hospital teaching status, hospital ownership, hospital region, income
Ong et al (2009)	age, gender, comorbidity, race, diagnosis, Medicare eligibility, hospital teaching status, hospital ownership, hospital location, hospital size, hospital procedure volume
Yasunaga et al (2009)	age, gender, BMI, diagnosis, comorbidity, hospital procedure volume
Paterson et al (2010)	age, gender, comorbidity, diagnosis, hospital teaching status, hospital procedure volume
Wei et al (2010)	age, gender, diagnosis, comorbidity, hospital ownership, hospital region
Styron et al (2011)	age, gender, race, comorbidity, income, insurance status, geographic region, hospital region, hospital teaching status, hospital ownership, hospital size, hospital procedure volume
Baker et al (2011)	age, surgeon volume, preoperative hemoglobin, gender, type of anaesthetic, ASA, surgeon experience, indication

(*ASA* American Society of Anesthesiologists, *BMI* Body mass index, *WOMAC* Western Ontario and McMaster Universities Osteoarthritis Index).

### Quality appraisal of studies

All 11 studies were observational studies and are considered low quality studies as per GRADE system guidelines. None of the studies warranted an upgrade to moderate quality due to the relative small effect of surgeon volume on outcomes examined.

### Study results

#### Mortality rate (MR) (In hospital - 2 studies, 90 day - 3 studies)

None of the studies identified a statistically significant relationship between surgeon volume and MR.

#### Pulmonary embolism and deep venous thrombosis (2 studies)

Among the 2 studies examining PE and/or DVT, neither reported a statistically significant association with surgeon volume.

#### Surgical site infection (5 studies)

Three of the 5 studies found a statistically significant association (p < 0.05) between low surgeon volume and infection rates [8,26,30]. Wei et al found a higher in-hospital infection rate among low volume (LV) surgeons [0.99% (LV) vs. 0.54% high volume (HV) surgeon, adjusted OR: 2.31 (CI: 1.379 - 3.876)] [26] and Muilwijk et al found a lower infection rate 1 year post-TKA among HV surgeons [2.1% (HV) vs 4.9% (LV), adjusted OR: 0.43 (CI: 0.23-0.80)] [30]. Neither study specified whether this was deep or superficial surgical site infection. Katz et al found decreased deep infections at 90 days post-TKA among HV surgeons [0.29% (HV) vs. 0.55% (LV), adjusted OR: 0.62 (CI: 0.37-1.06)], which was statistically significant for trend (p = 0.006) [8]. Two studies did not find a significant association between surgeon volume and infection rate [27,32].

#### Medical complications (6 studies)

Katz et al (2004) found a statistically significant decrease in pneumonia rates following TKA for HV surgeons [1.02% (HV) vs. 1.68% (LV), adjusted OR 0.72 (CI 0.54 - 0.95)] [8]. Baker et al (2011) found a statistically significant decrease in transfusion rate following TKA for HV surgeons compared to LV surgeons (4% vs 13%). The remaining 4 studies did not report a statistically significant association between surgeon volume and postoperative medical complications [25-27,32] (Table 2).

#### Length of stay (5 studies)

Four studies reported a significant increase in LOS among patients of LV surgeons [21,25,32,45]. Yasunaga et al did not find a significant association between LOS and surgeon volume [27]. Numerical data for LOS were not uniformly reported across studies and are summarized in Table 2.

#### Procedure length (one study)

Ong et al found a significant increase in TKA procedure time for LV surgeons (165 min vs 135 min) [28].

#### Implant survivorship (3 studies)

Manley et al (2009) did not find a significant association between 8-year implant survivorship and surgeon volume [18]. Similarly, Kreder et al (2003) and Paterson et al (2010) did not find a significant association between surgeon volume and 3-year and 1-year revision rate, respectively [25,32] (Table 2).

#### Patient-reported outcomes (one study)

Patients of LV surgeons had significantly lower (i.e. worse) WOMAC scores [76 (LV) vs. 83 (HV)], and were more likely to have a WOMAC score of < 60 [22.6% (LV) vs. 8.4% (HV), adjusted OR 2.1 (CI 1.1 - 4.2)], two years post TKA [9]. In addition, patients of LV surgeons were more likely to report an inability to flex the knee to 90 degrees (adjusted OR 2.9, CI 1.6 - 5.5), and more likely to report an inability to achieve full extension at 2 years post operation (adjusted OR 2.2, CI 1.1 - 4.4) [9] (Table 2).

## Discussion

Understanding the relationship between provider volume and outcomes for TKA is critical to informing discussions concerning ‘centralization’ or ‘regionalization’ [24] and overall efforts to improve quality and outcomes of care in TKA. The principle behind centralization or regionalization is that improved patient outcomes can be achieved by concentrating complex surgical procedures in regional centers, or “centers of excellence”. Several studies have demonstrated a relationship between higher provider volumes and improved outcomes for surgical procedures such as coronary artery bypass grafting, aortic aneurysm repair, carotid endarterectomy, and complex gastrointestinal surgeries [46]. Two principal hypotheses have been put forward to explain these observations: (1) physicians and hospitals develop more effective skills as they treat more patients (‘practice makes perfect’) and (2) physicians and hospitals reporting better outcomes receive more referrals and thus accrue larger volumes (‘selective referral’) [14,46]. If these hypotheses are broadly applicable, our expectation was that similar positive associations between higher surgeon volume and improved patient outcomes would be observed in TKA.

Our review of the limited literature in primary TKA revealed significant variability across studies in the categorization of surgeon procedure volumes. This variability rendered comparisons of study findings challenging, and highlighted a critical need for consistency in future research. Despite these challenges, we report a general trend of improved outcomes among ‘higher’ volume surgeons although we are careful to note that statistical significance was not achieved across all studies or outcomes. After reviewing the available studies, we would identify a high surgeon volume as > 50 TKA per year. Three studies identified a statistically significant relationship between low surgeon volume and higher infection rates (0.26% - 2.8% higher). While the magnitude of the reported differences in infection rates were relatively small, when considered in the context of the expected 3.5 million TKA procedures to be performed over the next 20 years in the US alone, this may represent a significant outcome at a population level.

The majority of studies examining LOS found a statistically significant increase in LOS for LV surgeons. Evidence on process of care adherence (such as use of antibiotic prophylaxis, DVT prophylaxis, beta-blocker use in high risk patients) has demonstrated that adherence to evidence-based processes of care improves quality of care and decreases LOS in total joint replacement surgery [23]. Bozic et al found a weak negative correlation between surgeon volume and number of missed evidence based processes of care [23]. It is possible that adherence to evidence based processes of care may account for some of the decreases in LOS for HV surgeons. Similarly, utilization of clinical care pathways for patients undergoing total joint arthroplasty have also demonstrated improved quality of care and shorter LOS [47-49]. It is not known however, if LV surgeons are less likely to utilize a clinical care pathway. Further study in the relationship between surgeon volume, clinical care pathways and process of care measures may be useful in understanding why LV surgeons have longer LOS.

Early to midterm (up to 8 years) implant survivorship did not appear to be influenced by surgeon volume [18,25,32]. Whether similar findings hold over the longer-term (i.e. >8 years) is unknown. Further long term survivorship studies would be useful in understanding the relationship between surgeon volume and implant survivorship.

In the one study which examined patient-reported outcomes, Katz et al (2007) reported a positive association between LV surgeons and poorer TKA outcomes [9]. However, the lack of additional studies examining patient-reported outcomes precludes any general statement concerning the influence of surgeon volume on these outcomes. It also highlights an important need for further work in this area to critically inform relevant policy discussions and decisions concerning TKA.

Studies examining hospital volume and TKA outcomes have demonstrated decreased mortality, infection and pulmonary embolism [8,10,16,32,39]. Furthermore, two studies have demonstrated decreased TKA survivorship for low volume hospitals [18,32]. Given these findings, hospital volume appears to have more impact on outcome for TKA than surgeon volume, however the quality and limitations of these studies prevents the delivery of definitive or ‘strong’ recommendations at this point [20]. Further studies will be important in determining the relative importance of surgeon volume and hospital volume to TKA outcome.

In 2002, Halm et al performed a review of the literature on volume-related outcomes in health care, and included a wide variety of surgical procedures [14]. They identified one study that examined the influence of hospital volume on TKA outcomes. In 2007, Shervin et al performed a review of orthopedic procedures, examining the association between hospital and surgeon volume, and outcomes [50]. In their review, they included 4 studies examining TKA outcomes and surgeon volume which are also included in this review. Finally, 2 recent reviews by Marlow et al and Critchely et al, examined surgeon volume and outcomes in TKA procedures. Both reviewed primary and revision knee arthroplasty procedures [24,51]. Marlow et al included 3 studies that examined surgeon volume and primary TKA outcomes [24]. Critchley et al presented data on primary hip and knee arthroplasty procedures together [51]. The present review differs from and expands on the prior reviews in that the present focus was on primary TKA outcomes alone, and the association with surgeon volume. Furthermore, we included several recently published studies not included in previous reviews.

Due to the nature of the literature from which this review was derived, there are study limitations. Studies on surgeon volume are primarily retrospective in nature and based on national health care databases and surveys which are limited in the number of variables collected. Furthermore, different health care databases capture different patient groups making comparisons challenging. Medicare data in the US contains data on patients over 65, which is different from other national health care databases such as OHIP. As with all non-randomized studies, there is potential for uncontrolled confounding factors which can bias the results. Additionally, the studies reviewed were variable in methodology, with variable thresholds for surgeon volume categorization. Similarly, studies were highly variable in outcomes measured (i.e. superficial vs. deep infection), and timing of measured outcomes (i.e. in-hospital vs. 90 day MR). The variability across the key variable of surgeon volume precluded formal meta-analysis from being performed.

While our findings suggest a trend towards better outcomes for higher volume surgeons, we do not believe there is sufficient evidence to fully support initiatives to concentrate procedures within specific regions or centres on the basis of surgeon volume alone. All of the available studies are of ‘low quality’ as per GRADE system, making any clinical recommendations challenging. In addition, it is unclear if this general trend of improved outcomes for higher volume surgeons warrants regionalization to higher volume surgeons. Studies examining the effect of regionalization of TKA to high volume hospitals provide some evidence that regionalization may not be the solution [35,52]. Evidence suggests that some patients would refuse to have surgery in an unfamiliar setting, preferring to attend a local health provider with lower procedure volume [35,52]. In the US, the poor, less educated, elderly, as well as racial/ethnic minorities are more likely to undergo TKA at low volume centers [11,36,38]. Regionalization of TKA to high volume centres and surgeons may further exacerbate existing disparities in the utilization of TKA and restrict access to some patients who would otherwise use a low volume provider for TKA, increasing the number of patients who decline or defer their elective TKA surgery with resultant poorer health outcomes [2,11,53]. A regionalization program involving referral to high volume surgeons and hospitals might decrease the already low rate of perioperative complications at the cost of increasing arthritis related disability [11]. Evidence suggests that having TKA in low volume hospitals costs more and produces worse outcomes than having TKA in high volume centers, but having TKA in low volume centers is still more cost effective than not having TKA at all [35]. While these studies were specific to examining the role of hospital volume on cost effectiveness of TKA, it is possible that the same may hold true for surgeon volume and TKA. Further study into the cost effectiveness of regionalization will be paramount before a universal referral program designed to shift patients to high volume surgeons becomes policy. Cost utilization and effectiveness will be crucial as healthcare resources become more scarce, with the principle of providing optimum patient care in the most efficient manner the ultimate goal.

## Conclusions

There is a general trend towards better outcomes for higher volume surgeons, however it is unclear if this trend warrants widespread adoption of regionalization policies. The evidence that is available is of ‘low quality’ and the strength of recommendations from the available evidence is weak. Further study is needed before regionalization policies are adopted.

## Abbreviations

TKA: Total knee arthroplasty; THA: Total hip arthroplasty; N: Sample size; US: United States; HCUP-NIS: Health Care Cost and Utilization Project Nationwide Inpatient Sample; OHIP: Ontario Health Insurance Plan; CIHI: Canadian Institute for Health Information; NHI: National Health Insurance; PREZIES: Preventie Ziekenhuisinfecties door Surveillance Network database; MR: Mortality rate; PE: Pulmonary embolism; DVT: Deep venous thrombosis; WOMAC: Western Ontario and McMaster Universities Osteoarthritis index; LOS: Length of stay; OR: Odds ratio; LV: Low volume; HV: High volume.

## Competing interests

The authors declare that they have no competing interests.

## Authors’ contributions

RLL: Literature search and abstract review. Data abstraction and data analysis. Manuscript drafting, approval of the final manuscript. AVP: Statistical analysis, critical manuscript review and editing, approval of the final manuscript. RG: Data abstraction and analysis, manuscript writing, approval of the final manuscript. NNM: Initial study conception, manuscript editing, approval of the final manuscript.

## Pre-publication history

The pre-publication history for this paper can be accessed here:

http://www.biomedcentral.com/1471-2474/13/250/prepub
